# The effect of the COVID-19 pandemic on life expectancy in 27 countries

**DOI:** 10.1038/s41598-023-35592-9

**Published:** 2023-06-01

**Authors:** Guogui Huang, Fei Guo, Klaus F. Zimmermann, Lihua Liu, Lucy Taksa, Zhiming Cheng, Massimiliano Tani, Marika Franklin

**Affiliations:** 1 Centre for Health Systems and Safety Research, Australian Institute of Health Innovation, Macquarie University, Level 6, 75 Talavera Road, Sydney, NSW 2109 Australia; 2 Department of Management, Macquarie Business School, Macquarie University, Sydney, Australia; 3 Global Labor Organization (GLO), Essen, Germany; 4 Department of Population and Public Health Sciences, Keck School of Medicine, University of Southern California, Los Angeles, USA; 5 Deakin University Business School, Deakin University, Burwood, Australia; 6 Social Policy Research Centre, University of New South Wales, Sydney, Australia; 7 School of Business, University of New South Wales, Canberra, Australia

**Keywords:** Epidemiology, Epidemiology, Viral infection

## Abstract

The expected year-on-year intrinsic mortality variations/changes are largely overlooked in the existing research when estimating the effect of the COVID-19 pandemic on mortality patterns. To fill this gap, this study provides a new assessment of the loss of life expectancy caused by COVID-19 in 27 countries considering both the actual and the expected changes in life expectancy between 2019 and 2020. Life expectancy in 2020 and the expected life expectancy in the absence of COVID-19 are estimated using the Lee-Carter model and data primarily from the Human Mortality Database. The results show that life expectancy in 21 of the 27 countries was expected to increase in 2020 had COVID-19 not occurred. By considering the expected mortality changes between 2019 and 2020, the study shows that, on average, the loss of life expectancy among the 27 countries in 2020 amounted to 1.33 year (95% CI 1.29–1.37) at age 15 and 0.91 years (95% CI 0.88–0.94) at age 65. Our results suggest that if the year-on-year intrinsic variations/changes in mortality were considered, the effects of COVID-19 on mortality are more profound than previously understood. This is particularly prominent for countries experiencing greater life expectancy increase in recent years.

## Introduction

Since its outbreak at the end of 2019, the ongoing COVID-19 pandemic has evolved into the largest public health crisis of the new millennium. As of 5 August 2022, COVID-19 had spread to almost all countries and territories globally, infecting more than 585 million people and causing more than six million deaths^1^. Given the insufficient testing and the under-reporting of cases and deaths in many countries, the actual number of deaths from COVID-19 is still believed to be considerably underestimated. Additionally, inconsistencies in public health systems globally in defining and classifying COVID-19 deaths, and the indirect effects of the pandemic on other causes of death, make it difficult to estimate an accurate death toll of the pandemic^2–5^. As the COVID-19 pandemic is still ongoing and is causing profound challenges for humanity, it is vital to better understand the pandemic’s effects on mortality, including its relevant characteristics and patterns.

A large number of studies tend to estimate the effect of COVID-19 on mortality by measuring life expectancy in 2020 and comparing the changes to life expectancy between 2019 and 2020^2,3,6–12^. The basic principle of this strategy is that, assuming no other new major events exerted a significant effect on mortality in 2020, the differences in mortality levels between 2019 and 2020 can be attributed to the COVID-19 pandemic. The changes to mortality caused by COVID-19 can be summarised through the differences in life expectancy between 2019 and 2020, given that life expectancy is a summary indicator of the age-specific death rate and is not affected by a population’s size or age structure. Since life expectancy is age-standardised, it is comparable between two periods within one population and between two populations for a similar period^13^; hence, it can be used to compare the observed mortality levels with the consideration of COVID-19 and in the absence of COVID-19 across time and among different countries. Following this strategy, existing studies find that the COVID-19 pandemic has caused substantially elevated mortality in 2020, notably lowering life expectancy between 2019 and 2020 or between 2018 and 2020 in many countries, particularly those in Europe and the Americas^6,10,14^. In addition, the decrease in life expectancy varied considerably according to sex and race, being disproportionally higher among men^9^ and in racial minority groups^10,11^.

**Figure 1:**
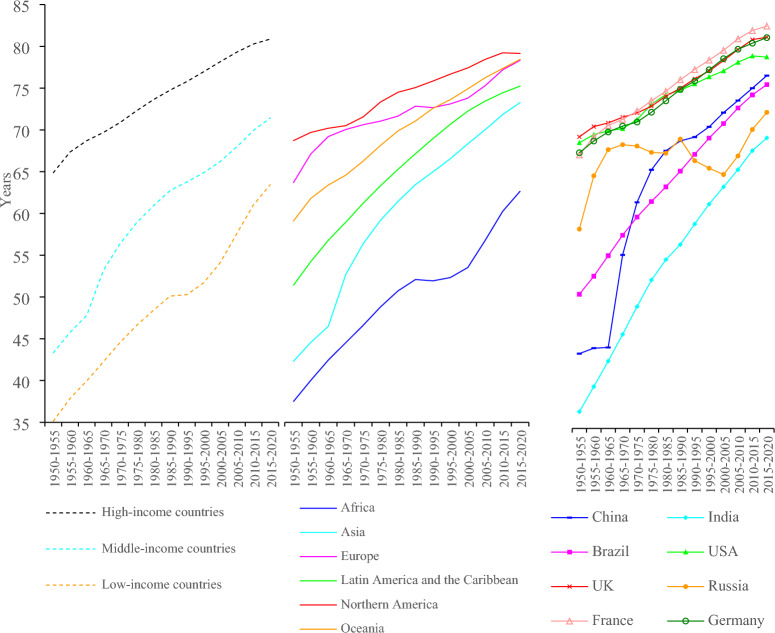
Changes in life expectancy worldwide and in major countries between 1980 and 2020. *Data Source*: World Population Prospects.

However, simply tracking the actual changes in life expectancy between 2019 and 2020, a method used in many existing studies, may not fully capture the effects of COVID-19 on mortality because this strategy overlooks the year-on-year intrinsic variations of mortality over time in the absence of COVID-19^15^. This is the so-called counter-factual: what would have happened in 2020 without the COVID-19 outbreak. It is well established that since the end of World War II, most countries have experienced profound demographic transitions, with continuously increasing life expectancy over more than seven decades. The increasing trajectories of life expectancy have not been linear: there have been varying paces across nations and even occasional setbacks during specific periods for certain countries (see Fig. 1). The overall increase in life expectancy was expected to continue in 2020 in almost all countries had COVID-19 not occurred^16^. However, the outbreak of COVID-19 pandemic reversed this upward trend in 2020^2,9^. Consequently, any potential gain in life expectancy that year is expected to have been eliminated. Therefore, without considering the expected year-on-year changes in mortality in 2020, the full loss of life expectancy caused by the COVID-19 pandemic would be possible highly underestimated.

This study presents a new assessment of the loss of life expectancy caused by COVID-19 in 27 countries, providing an improved approach in measuring the effects of sudden and profound events on mortality changes. We apply the Lee–Carter model to project the trajectory-of-life expectancy in 2020, using it as a new baseline to measure COVID-19’s effects on mortality. The Lee–Carter model is a powerful tool to project mortality trajectory but has not been adequately used in assessing COVID-19-induced mortality variations. Using this model, this study considers both the actual and the expected changes in life expectancy between 2019 and 2020, providing a more comprehensive picture of COVID-19’s effects on mortality. We avoid predicting 2021 to limit model prediction errors. Our results have important implications in relation to how we better assess and understand COVID-19’s devastating effects on human lives and the profound challenges caused by future pandemics and other major events.

## Data and methods

### Data

This study uses data from the Human Mortality Database^17^, the Eurostat^18^ and the Office for National Statistics (United Kingdom)^19^ to estimate and analyse life expectancy during 1990 to 2020 in 27 countries (Australia, Austria, Belgium, Bulgaria, Canada, Chile, the Czech Republic, Denmark, England and Wales, Finland, France, Germany, Greece, Hungary, Japan, Ireland, Italy, the Netherlands, Norway, Poland, Portugal, Scotland, Slovakia, Spain, Sweden, Switzerland and the United States [US]). These countries are chosen given data availability and that they represent well the countries first reached by the COVID-19 pandemic outside China. Specifically, data on the population size and age structure in 2020 for England and Wales and Scotland are derived from the Office for National Statistics, and data on the population size and age structure in 2020 for the other 20 European countries are extracted from the Eurostat. Information on population size and age structure in 2020 for Australia, Canada, Chile, Japan, and the US are drawn from the Human Mortality Database. Mortality data in 2020 for all 27 countries are obtained from the Human Mortality Database Short-Term Mortality Fluctuations (STMF) files (except Australia, Canada, Germany, Japan and Ireland, of which mortality data in 2020 were drawn from Human Mortality Database directly). The Human Mortality Database STMF files provide the weekly number of deaths in 38 countries or regions, among which Iceland and Luxembourg are excluded given the small population size, Israel, New Zealand and Northern Ireland are excluded given too wide an age interval of data (e.g., 20-year age group or greater), while Croatia and South Korea are excluded because of insufficient historical mortality data in the Human Mortality Database. Additionally, Estonia, Latvia, Lithuania and Slovenia are excluded because their historical data of crude death rates contained many zeros at certain ages, which is not accepted by the Lee–Carter model used in this study. The remaining two countries or regions (i.e., Chinese Taiwan and Russia) are excluded because of incomplete mortality data for 2020 in the STMF dataset at the time this study was conducted. Historical data on the crude death rate and the population size for all 27 countries from 1990 (except Chile from 1992), which are used to project the mortality level in 2020, are also obtained from the Human Mortality Database.

### Methods

#### Estimate of actual life expectancy in 2020

This study constructs life tables for the 27 countries following the method proposed by Chiang^20^. Abridged life tables of the US in 2020 are constructed with eight age groups (10-year age groups from 15–24 years to 75–84 years, and 85 years and older), and abridged life tables for the remaining 26 countries in 2020 are constructed with 16 age groups (5-year age groups from 15–19 years to 85–89 years, and 90 years and older). Complete life tables for the years prior to 2020, with 90 years and older as the last open-ended age group, for all 27 countries are constructed using mortality data from the Human Mortality Database. Given that data on the death number at age 0 and for ages 1–4 are unavailable in 20 of the 27 countries, and that the case fatality rate of COVID-19 is negligible under age 15^21,22^, this study estimated life expectancy at age 15 and age 65 for both men and women. Age 15 is chosen since it is the starting age of many crucial indicators, such as labour force participation rate and fertility, while age 65 is chosen given that it is widely used to define the elderly population. Measuring the changes of life expectancy at these two ages can assist to better capture the effect of COVID-19 on mortality among the adult populations and the elderly populations. Full explanations of the construction procedures of these life tables are presented in the Supplementary Information to this study.

#### Projection of life expectancy in 2020

This study uses the Lee-Carter model proposed by Lee and Carter^23^ to project life expectancy in 2020. The Lee–Carter model is widely used in forecasting mortality based on historical mortality trends, with various extensions suggested by different scholars^24^. The popularity of this model is primarily due to its capacity to capture the major variability of past mortality data, its effective interpretations of model parameters and its minimal use of subjective judgement^25^. The Lee–Carter model has been applied in forecasting future mortality rate in many countries, including the US^26,27^, England and Wales^28^, Sweden^29^, Australia^30^ and New Zealand^31^. In this study, life expectancy in 2020 projected by the Lee-Carter model, rather than the observed life expectancy in 2019 (commonly used in the existing studies), is used as the benchmark to measure the loss of life expectancy instigated by COVID-19 in 2020. This better captures the loss of life expectancy caused by COVID-19 as it considers both the actual decrease in life expectancy observed in 2020 and the possible variations in life expectancy in the absence of COVID-19.

The basic form of the Lee–Carter model is presented as follows:

$$ln\, m_{x,t} = a_{x} + b_{x} *k_{t} + \varepsilon_{x,t}$$

here $m_{x,t}$ is the central mortality rate at age *x* at time *t*; $a_{x}$ is the overall pattern of mortality; $b_{x}$ measures the sensitivity of $ln m_{x,t}$ to the changes of $k_{t}$ over time; $k_{t}$ represents the time trend of the general mortality; and $\varepsilon_{x,t}$ is the residual.

This study uses the three-decade long historical mortality data (from 1990 onwards) to estimate $$a_{x}$$, $$b_{x}$$ and $$k_{t}$$ for all 27 countries (except Chile which was based on data since 1992). The singular value decomposition approach is applied to obtain unique estimates for $$b_{x}$$ and $$k_{t}$$, subject to two constraints with $$b_{x}$$ sum to 1 and $$k_{t}$$ sum to 0, while $$a_{x}$$ is obtained by averaging $$ln m_{x,t}$$. The computation of the singular value decomposition is conducted using the command of ‘*leecart*’ in Stata (version 17.0, StataCorp LP, College Station, Texas 77,845 USA, the same hereafter). Then, the estimated $$k_{t}$$ is modelled as a random walk with trend and was projected using the Auto Regressive Integrated Moving Average (ARIMA) time series model, forecasting variables using the series past values; this has been applied by many prior studies to project the trajectory of $$k_{t}$$ ^31,32^. While the Lee-Carter model captures the long-term mortality trend based on three-decade historical mortality data, the use of ARIMA random walk model helps capture the short-term changes in mortality given the capacity of the ARIMA random walk model in capturing short-term non-linearities. The computation of ARIMA model was conducted using the command of ‘*arima*’ in Stata, and the determination approaches of the coefficients (p, d, p) of ARIMA are described in the Supplementary Information. After $$k_{t}$$ is modelled and $$k_{2020}$$ is projected, the expected $$m_{x,2020}$$ is estimated by taking exponentials of ($$a_{x} + b_{x} *k_{2020} )$$, after which life expectancy in 2020 in the absence of COVID-19 can be estimated.

## Results

**Figure 2:**
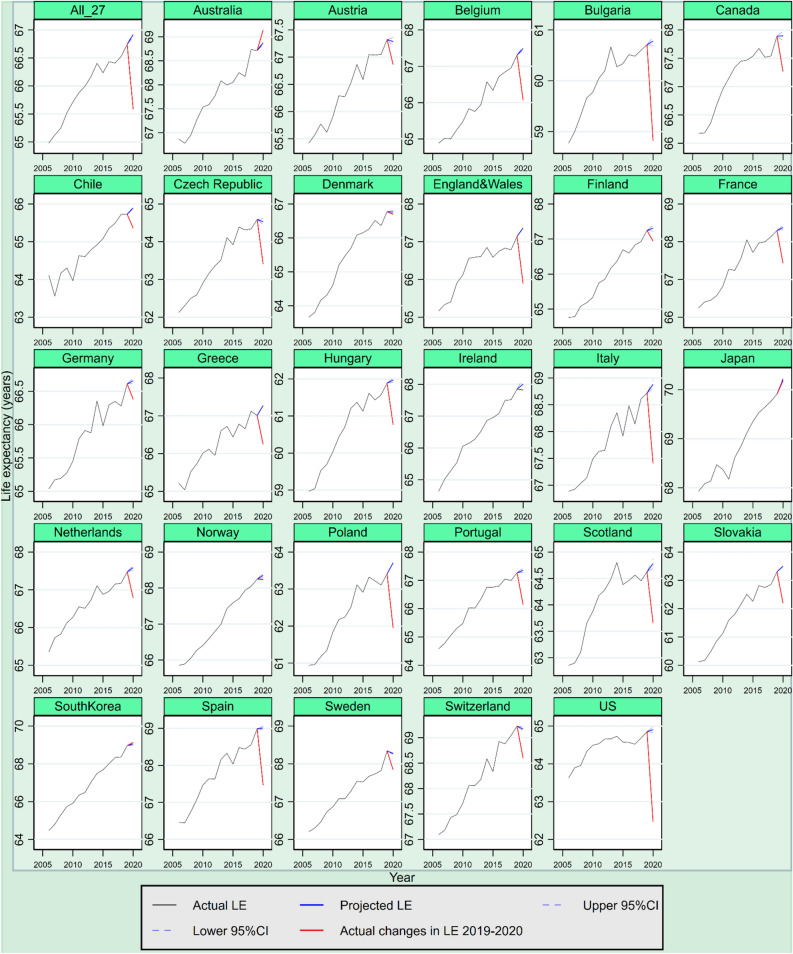
Actual and expected life expectancy (LE) at age 15—effects of the COVID-19 pandemic in 27 countries. Actual life expectancy at age 15 before the COVID-19 pandemic is indicated by solid black line and actual life expectancy at 15 from 2019 to 2020 is indicated by red lines. Projected life expectancy at age 15 (i.e., in absence of COVID-19) is indicated by blue line, with 95% confidence intervals (CI) indicated by dashed blue line.

**Figure 3:**
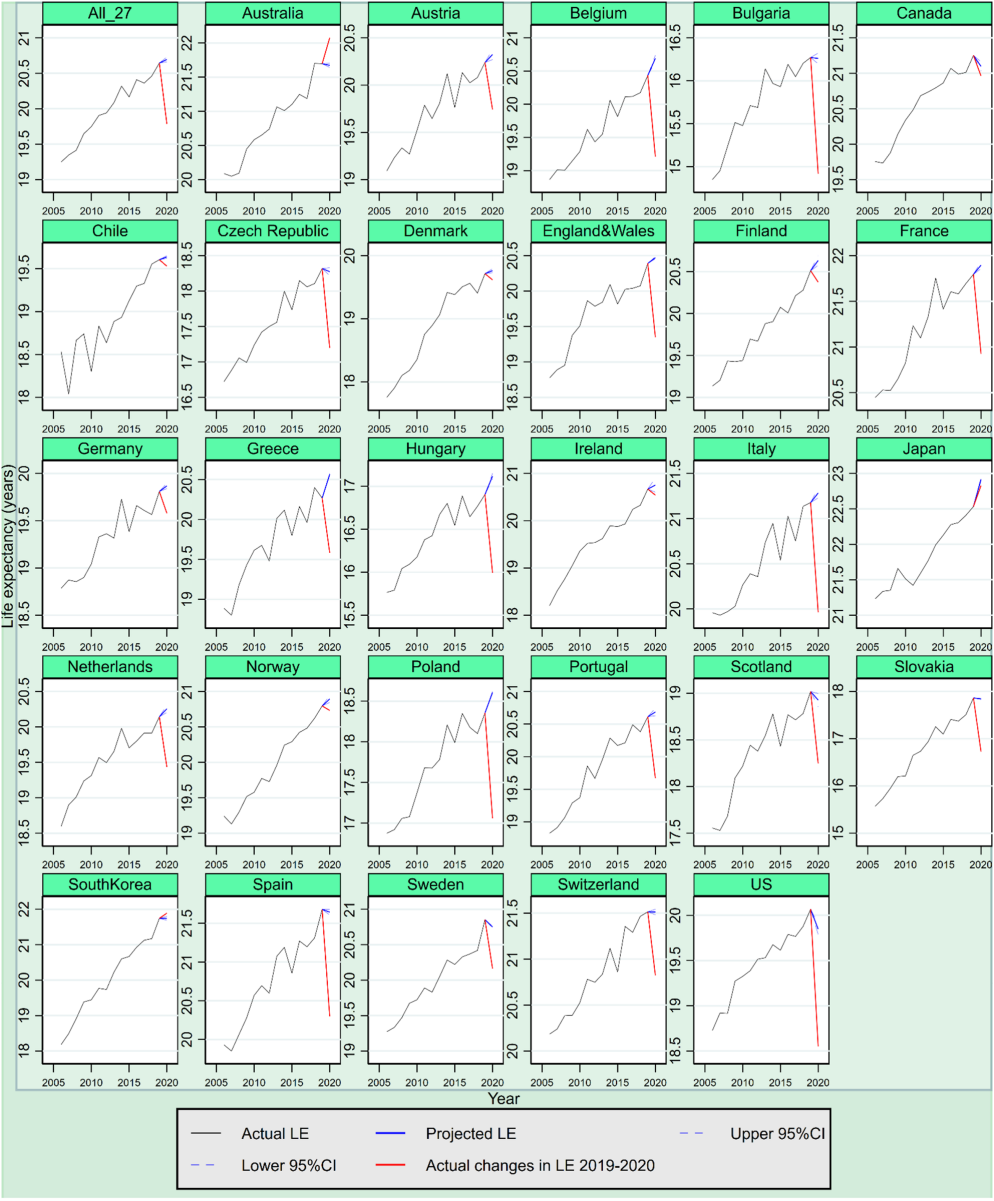
Actual and expected life expectancy (LE) at age 65—effects of the COVID-19 pandemic in 27 countries. Actual life expectancy at age 65 before the COVID-19 pandemic is indicated by solid black line and actual life expectancy at 65 from 2019 to 2020 is indicated by red lines. Projected life expectancy at age 65 (i.e., in absence of COVID-19) is indicated by blue line, with 95% confidence intervals (CI) indicated by dashed blue line.

The projection results show that in the absence of COVID-19, life expectancy was expected to increase by varying degrees between 2019 and 2020 in most of the 27 countries. To be precise, life expectancy at age 15 among 21 out of the 27 countries was expected to increase in 2020 had COVID 19 not occurred, from 0.01 years (95% CI =  − 0.08 years, 0.09 years) in Canada to 0.30 years (95% CI = 0.27 years, 0.33 years) in Japan (see Fig. 2). Life expectancy at age 65 among 17 out of the 27 countries was also expected to grow that year, from 0.03 years (95% CI = 0.01 years, 0.04 years) in Chile to 0.39 years (95% CI = 0.36 years, 0.40 years) in Japan, assuming that the COVID-19 had not occurred (see Fig. 3). Consequently, after using the Lee-Carter model to consider the expected year-on-year intrinsic changes to life expectancy, the loss of life expectancy due to COVID-19 among the 27 countries is better captured, amounting to 1.33 year (95% CI = 1.29 years, 1.37 years) at age 15 on average, instead of 1.16 years without considering the intrinsic mortality variations, and to 0.91 years (95% CI = 0.88 years, 0.94 years) at age 65 on average, instead of 0.82 years without the intrinsic mortality variations being considered.

After considering both the actual and the expected changes and intrinsic variations to life expectancy between 2019 and 2020, the results show that the largest loss of life expectancy at age 15 among the 27 countries was observed in the US (− 2.33 years, 95% CI =  − 2.41 years, − 2.26 years), followed by Bulgaria (− 1.96 years, 95% CI =  − 2.05 years, − 1.88 years), Poland (− 1.76 years, 95% CI =  − 1.77 years, − 1.74 years), Spain (− 1.55 years, 95% CI =  − 1.60 years, − 1.50 years) and Italy (− 1.48 years, 95% CI =  − 1.55 years, − 1.40 years) (see Table 1). In these five countries, life expectancy at age 15 regressed to a level not seen since 1998, 2007, 2009, 2010 and 2009, respectively. Additionally, loss of life expectancy at age 65 was notable across all 27 countries, with the five highest figures observed in Poland (− 1.55 years, 95% CI =  − 1.56 years, − 1.54 years), Belgium (− 1.48 years, 95% CI =  − 1.53 years, − 1.43 years), Spain (− 1.35 years, 95% CI =  − 1.38 years, − 1.31 years), Bulgaria (− 1.34 years, 95% CI =  − 1.41 years, − 1.28 years) and Italy (− 1.32 years, 95% CI =  − 1.37 years, − 1.27 years) (see Table 2). In these five countries, life expectancy at age 65 regressed to a level not seen since 2007, 2010, 2009, 2006 and 2008, respectively.

**Table 1:** Actual life expectancy at age 15 ($LE_{A,15}$), expected life expectancy at age 15 ($LE_{E,15}$) and their gaps in 2020 in 27 countries.

	Total			Female			Male		
	$LE_{A,15}$	$LE_{E,15}$(95% CI)	$LE_{A,15}$ − $LE_{E,15}$ (95% CI)	$LE_{A,15}$	$LE_{E,15}$(95% CI)	$LE_{A,15}$ − $LE_{E,15}$ (95% CI)	$LE_{A,15}$	$LE_{E,15}$(95% CI)	$LE_{A,15}$ − $LE_{E,15}$ (95% CI)
Australia	69.15	68.87 (68.83,68.91)	0.27 (0.23,0.31)	71.18	70.88 (70.85,70.91)	0.30 (0.27,0.33)	67.11	66.92 (66.86,66.99)	0.18 (0.12,0.25)
Austria	66.86	67.29 (67.21,67.36)	− 0.43 (− 0.50, − 0.35)	69.12	69.40 (69.34,69.45)	− 0.28 (− 0.33, − 0.23)	64.55	64.90 (64.83,64.96)	− 0.35 (− 0.42, − 0.29)
Belgium	66.06	67.49 (67.43,67.55)	− 1.43 (− 1.49, − 1.36)	68.31	69.64 (69.63,69.65)	− 1.33 (− 1.34, − 1.32)	63.81	65.34 (65.25,65.43)	− 1.53 (− 1.61, − 1.44)
Bulgaria	58.82	60.78 (60.69,60.87)	− 1.96 (− 2.05, − 1.88)	62.67	64.53 (64.32,64.75)	− 1.86 (− 2.08, − 1.65)	55.24	57.49 (57.47,57.50)	− 2.25 (− 2.27, − 2.23)
Canada	67.26	67.89 (67.81,67.98)	− 0.63 (− 0.72, − 0.55)	69.49	70.06 (69.99,70.12)	− 0.56 (− 0.63, − 0.49)	65.03	65.94 (65.86,66.03)	− 0.91 (− 0.99, − 0.83)
Chile	65.36	65.90 (65.87,65.92)	− 0.54 (− 0.56, − 0.52)	68.29	68.48 (68.45,68.51)	− 0.19 (− 0.22, − 0.16)	62.36	63.36 (63.36,63.37)	− 1.00 (− 1.01, − 1.00)
Czech Republic	63.41	64.53 (64.45,64.61)	− 1.12 (− 1.20, − 1.04)	66.40	67.42 (67.35,67.49)	− 1.02 (− 1.09, − 0.94)	60.48	61.68 (61.62,61.74)	− 1.20 (− 1.26, − 1.15)
Denmark	66.70	66.77 (66.72,66.81)	− 0.07 (− 0.12, − 0.03)	68.63	68.61 (68.59,68.63)	0.02 (0.00,0.04)	64.75	64.74 (64.70,64.79)	0.01 (− 0.04,0.06)
England & Wales	65.89	67.36 (67.34,67.38)	− 1.48 (− 1.50, − 1.46)	67.83	68.99 (68.93,69.05)	− 1.16 (− 1.22, − 1.10)	63.96	65.62 (65.54,65.69)	− 1.65 (− 1.73, − 1.58)
Finland	66.94	67.31 (67.23,67.39)	− 0.37 (− 0.45, − 0.29)	69.70	70.07 (69.85,70.29)	− 0.37 (− 0.59, − 0.15)	64.18	64.56 (64.50,64.61)	− 0.38 (− 0.43, − 0.33)
France	67.43	68.38 (68.32,68.43)	− 0.95 (− 1.00, − 0.90)	70.35	71.19 (71.14,71.24)	− 0.85 (− 0.90, − 0.80)	64.43	65.35 (65.28,65.41)	− 0.92 (− 0.98, − 0.85)
Germany	66.38	66.66 (66.62,66.70)	− 0.28 (− 0.32, − 0.24)	68.79	68.98 (68.94,69.02)	− 0.19 (− 0.23, − 0.15)	63.97	64.30 (64.25,64.34)	− 0.32 (− 0.37, − 0.27)
Greece	66.24	67.27 (67.26,67.29)	− 1.03 (− 1.05, − 1.02)	68.73	69.79 (69.73,69.85)	− 1.06 (− 1.12, − 0.99)	63.77	64.70 (64.65,64.76)	− 0.93 (− 0.98, − 0.88)
Hungary	60.77	61.97 (61.92,62.03)	− 1.21 (− 1.26, − 1.15)	64.10	65.18 (64.82,65.54)	− 1.08 (− 1.44, − 0.72)	57.29	58.67 (58.56,58.77)	− 1.38 (− 1.48, − 1.27)
Ireland	67.83	68.01 (67.65,68.36)	− 0.18 (− 0.53,0.18)	69.63	70.04 (69.84,70.24)	− 0.41 (− 0.62, − 0.21)	66.03	65.86 (65.81,65.91)	0.18 (0.12,0.23)
Italy	67.41	68.88 (68.81,68.96)	− 1.48 (− 1.55, − 1.40)	69.70	71.01 (70.94,71.07)	− 1.31 (− 1.38, − 1.25)	65.04	66.55 (66.48,66.62)	− 1.51 (− 1.58, − 1.43)
Japan	70.18	70.21 (70.18,70.24)	− 0.03 (− 0.06,0.00)	73.32	73.50 (73.47,73.52)	− 0.17 (− 0.20, − 0.15)	66.94	66.78 (66.76,66.80)	0.16 (0.15,0.18)
Netherlands	66.79	67.59 (67.52,67.66)	− 0.80 (− 0.87, − 0.73)	68.43	69.01 (68.98,69.04)	− 0.59 (− 0.62, − 0.55)	65.14	66.03 (65.96,66.11)	− 0.89 (− 0.97, − 0.82)
Norway	68.24	68.35 (68.29,68.42)	− 0.12 (− 0.18, − 0.05)	69.91	70.10 (70.07,70.13)	− 0.19 (− 0.22, − 0.16)	66.53	66.82 (66.60,67.03)	− 0.29 (− 0.51, − 0.08)
Poland	61.95	63.71 (63.69,63.73)	− 1.76 (− 1.77, − 1.74)	66.15	67.30 (67.22,67.37)	− 1.15 (− 1.22, − 1.07)	57.87	59.88 (59.85,59.91)	− 2.01 (− 2.04, − 1.98)
Portugal	66.14	67.36 (67.28,67.43)	− 1.21 (− 1.29, − 1.14)	69.09	70.20 (70.18,70.22)	− 1.11 (− 1.13, − 1.09)	63.04	64.29 (64.14,64.44)	− 1.25 (− 1.40, − 1.10)
Scotland	63.66	64.78 (64.69,64.87)	− 1.12 (− 1.22, − 1.03)	65.90	66.58 (66.55,66.61)	− 0.68 (− 0.71, − 0.64)	61.40	62.80 (62.75,62.86)	− 1.40 (− 1.46, − 1.35)
Slovakia	62.19	63.50 (63.49,63.52)	− 1.31 (− 1.32, − 1.29)	65.56	66.70 (66.59,66.82)	− 1.14 (− 1.26, − 1.03)	58.76	59.81 (59.79,59.83)	− 1.05 (− 1.07, − 1.03)
South Korea	69.14	69.06 (68.98,69.13)	0.09 (0.02,0.16)	72.06	71.91 (71.84,71.97)	0.15 (0.09,0.22)	65.98	65.85 (65.76,65.94)	0.13 (0.04,0.22)
Spain	67.47	69.01 (68.96,69.06)	− 1.55 (− 1.60, − 1.50)	70.18	71.66 (71.61,71.71)	− 1.48 (− 1.53, − 1.43)	64.76	66.26 (66.21,66.32)	− 1.51 (− 1.56, − 1.45)
Sweden	67.84	68.27 (68.25,68.29)	− 0.43 (− 0.45, − 0.41)	69.50	69.70 (69.66,69.74)	− 0.20 (− 0.24, − 0.16)	66.18	66.53 (66.46,66.60)	− 0.36 (− 0.43, − 0.28)
Switzerland	68.60	69.17 (69.13,69.21)	− 0.57 (− 0.61, − 0.53)	70.52	70.93 (70.89,70.96)	− 0.41 (− 0.44, − 0.37)	66.63	67.48 (67.44,67.51)	− 0.85 (− 0.88, − 0.81)
US	62.47	64.80 (64.73,64.88)	− 2.33 (− 2.41, − 2.26)	65.26	67.39 (67.35,67.44)	− 2.14 (− 2.18, − 2.09)	59.78	62.29 (62.19,62.39)	− 2.51 (− 2.61, − 2.41)

*CI* Confidence interval.

**Table 2:** Actual life expectancy at age 65 ($LE_{A,65}$), expected life expectancy at age 65 ($LE_{E,65}$) and their gaps in 2020 in 27 countries.

	Total			Female			Male		
	$LE_{A,65}$	$LE_{E,65}$(95% CI)	$LE_{A,65}$ − $LE_{E,65}$ (95% CI)	$LE_{A,65}$	$LE_{E,65}$(95% CI)	$LE_{A,65}$ − $LE_{E,65}$ (95% CI)	$LE_{A,65}$	$LE_{E,65}$(95% CI)	$LE_{A,65}$ − $LE_{E,65}$ (95% CI)
Australia	22.08	21.67 (21.64,21.70)	0.40 (0.37,0.43)	23.38	23.01 (22.98,23.03)	0.38 (0.35,0.40)	20.70	20.33 (20.28,20.38)	0.37 (0.32,0.42)
Austria	19.74	20.32 (20.27,20.38)	− 0.58 (− 0.63, − 0.53)	21.22	21.61 (21.57,21.66)	− 0.39 (− 0.44, − 0.35)	18.08	18.64 (18.60,18.69)	− 0.56 (− 0.61, − 0.52)
Belgium	19.21	20.69 (20.65,20.74)	− 1.48 (− 1.53, − 1.43)	20.74	22.14 (22.14,22.15)	− 1.40 (− 1.41, − 1.40)	17.55	19.10 (19.04,19.17)	− 1.56 (− 1.62, − 1.49)
Bulgaria	14.92	16.26 (16.20,16.32)	− 1.34 (− 1.41, − 1.28)	16.83	18.16 (17.97,18.35)	− 1.33 (− 1.52, − 1.14)	12.72	14.33 (14.32,14.34)	− 1.61 (− 1.62, − 1.60)
Canada	20.96	21.10 (21.03,21.16)	− 0.14 (− 0.20, − 0.07)	22.29	22.57 (22.52,22.63)	− 0.29 (− 0.34, − 0.23)	19.53	19.69 (19.63,19.75)	− 0.16 (− 0.23, − 0.10)
Chile	19.53	19.64 (19.62,19.65)	− 0.11 (− 0.12, − 0.09)	21.30	21.11 (21.09,21.13)	0.19 (0.17,0.21)	17.50	18.02 (18.02,18.03)	− 0.52 (− 0.53, − 0.52)
Czech Republic	17.20	18.27 (18.22,18.33)	− 1.08 (− 1.13, − 1.02)	18.99	19.90 (19.84,19.96)	− 0.91 (− 0.97, − 0.85)	15.19	16.39 (16.35,16.42)	− 1.19 (− 1.23, − 1.16)
Denmark	19.62	19.76 (19.73,19.79)	− 0.15 (− 0.18, − 0.11)	20.97	21.03 (21.02,21.04)	− 0.06 (− 0.07, − 0.05)	18.18	18.35 (18.32,18.38)	− 0.17 (− 0.21, − 0.14)
England & Wales	19.35	20.47 (20.46,20.49)	− 1.12 (− 1.14, − 1.11)	20.59	21.50 (21.46,21.55)	− 0.91 (− 0.96, − 0.87)	18.05	19.28 (19.22,19.34)	− 1.24 (− 1.30, − 1.18)
Finland	20.37	20.63 (20.58,20.69)	− 0.26 (− 0.32, − 0.20)	21.93	22.28 (22.10,22.47)	− 0.35 (− 0.53, − 0.17)	18.60	18.78 (18.74,18.81)	− 0.17 (− 0.21, − 0.14)
France	20.93	21.90 (21.86,21.93)	− 0.97 (− 1.01, − 0.93)	22.81	23.67 (23.63,23.71)	− 0.87 (− 0.91, − 0.82)	18.82	19.74 (19.70,19.78)	− 0.92 (− 0.97, − 0.88)
Germany	19.58	19.87 (19.84,19.90)	− 0.29 (− 0.32, − 0.26)	21.14	21.36 (21.32,21.39)	− 0.22 (− 0.25, − 0.19)	17.87	18.20 (18.16,18.23)	− 0.32 (− 0.36, − 0.29)
Greece	19.58	20.56 (20.55,20.58)	− 0.98 (− 0.99, − 0.97)	20.99	21.83 (21.77,21.88)	− 0.84 (− 0.90, − 0.79)	18.08	19.10 (19.06,19.14)	− 1.01 (− 1.06, − 0.97)
Hungary	16.00	17.12 (17.09,17.15)	− 1.12 (− 1.16, − 1.09)	17.71	18.70 (18.45,18.95)	− 0.99 (− 1.24, − 0.74)	13.77	14.95 (14.90,15.00)	− 1.18 (− 1.23, − 1.13)
Ireland	20.54	20.75 (20.47,21.03)	− 0.21 (− 0.49,0.07)	21.81	22.18 (22.01,22.35)	− 0.37 (− 0.54, − 0.20)	19.23	19.18 (19.14,19.22)	0.06 (0.02,0.10)
Italy	19.96	21.28 (21.23,21.34)	− 1.32 (− 1.37, − 1.27)	21.62	22.83 (22.78,22.89)	− 1.22 (− 1.27, − 1.17)	18.15	19.45 (19.40,19.50)	− 1.30 (− 1.35, − 1.25)
Japan	22.83	22.92 (22.89,22.94)	− 0.09 (− 0.11, − 0.07)	25.27	25.45 (25.43,25.47)	− 0.18 (− 0.20, − 0.16)	20.12	20.03 (20.02,20.05)	0.08 (0.07,0.10)
Netherlands	19.44	20.26 (20.20,20.31)	− 0.82 (− 0.87, − 0.77)	20.68	21.41 (21.39,21.44)	− 0.73 (− 0.76, − 0.70)	18.13	18.93 (18.87,18.99)	− 0.80 (− 0.86, − 0.75)
Norway	20.73	20.89 (20.85,20.94)	− 0.16 (− 0.21, − 0.11)	21.88	22.09 (22.07,22.11)	− 0.21 (− 0.23, − 0.18)	19.46	19.70 (19.55,19.86)	− 0.24 (− 0.40, − 0.08)
Poland	17.06	18.61 (18.60,18.62)	− 1.55 (− 1.56, − 1.54)	19.22	20.30 (20.24,20.36)	− 1.08 (− 1.14, − 1.02)	14.49	16.23 (16.21,16.25)	− 1.74 (− 1.76, − 1.72)
Portugal	19.67	20.68 (20.63,20.74)	− 1.01 (− 1.07, − 0.96)	21.33	22.33 (22.31,22.35)	− 0.99 (− 1.01, − 0.98)	17.73	18.67 (18.56,18.77)	− 0.94 (− 1.04, − 0.83)
Scotland	18.25	18.87 (18.80,18.94)	− 0.62 (− 0.69, − 0.55)	19.37	19.78 (19.75,19.80)	− 0.41 (− 0.43, − 0.38)	17.03	17.74 (17.70,17.79)	− 0.72 (− 0.76, − 0.68)
Slovakia	16.73	17.84 (17.83,17.85)	− 1.12 (− 1.13, − 1.11)	18.48	19.52 (19.42,19.61)	− 1.04 (− 1.13, − 0.94)	14.53	15.47 (15.46,15.48)	− 0.94 (− 0.95, − 0.93)
South Korea	21.89	21.74 (21.69,21.80)	0.15 (0.09,0.20)	23.90	23.64 (23.58,23.69)	0.27 (0.21,0.33)	19.41	19.26 (19.19,19.32)	0.15 (0.09,0.22)
Spain	20.30	21.64 (21.61,21.68)	− 1.35 (− 1.38, − 1.31)	22.22	23.49 (23.45,23.54)	− 1.27 (− 1.32, − 1.23)	18.23	19.54 (19.50,19.57)	− 1.31 (− 1.34, − 1.27)
Sweden	20.16	20.75 (20.74,20.76)	− 0.59 (− 0.60, − 0.57)	21.36	21.84 (21.80,21.87)	− 0.48 (− 0.51, − 0.45)	18.90	19.46 (19.41,19.52)	− 0.56 (− 0.62, − 0.51)
Switzerland	20.82	21.51 (21.48,21.54)	− 0.69 (− 0.72, − 0.66)	22.19	22.78 (22.75,22.80)	− 0.59 (− 0.62, − 0.56)	19.33	20.18 (20.16,20.21)	− 0.86 (− 0.88, − 0.83)
US	18.55	19.67 (19.61,19.73)	− 1.12 (− 1.17, − 1.06)	19.80	21.11 (21.07,21.15)	− 1.31 (− 1.35, − 1.27)	17.19	18.19 (18.11,18.27)	− 1.00 (− 1.07, − 0.92)

*CI* Confidence interval.

Strikingly, Australia, Japan and two northern European countries (i.e., Denmark and Norway) experienced a significantly smaller loss of life expectancy caused by COVID-19 at both age 15 and age 65 compared with other nations (see Table 1). Notably, Australia even recorded a slight increase in life expectancy in 2020, with life expectancy at age 15 growing by 0.27 years (95% CI = 0.23 years, 0.31 years) and life expectancy at age 65 by 0.40 years (95% CI = 0.37 years, 0.43 years) that year. Japan, Denmark and Norway experienced almost no changes in life expectancy at age 15 (i.e., − 0.03 years, 95% CI =  − 0.06 years, 0.01 years for Japan; − 0.07 years, 95% CI =  − 0.12 years, − 0.03 years for Denmark; − 0.12 years, 95% CI =  − 0.18 years, − 0.05 years for Norway) and age 65 (i.e., − 0.09 years, 95% CI =  − 0.12 years, − 0.07 years for Japan; − 0.15 years, 95% CI =  − 0.18 years, − 0.12 years for Denmark; − 0.16 years, 95% CI =  − 0.21 years, − 0.11 years for Norway) in 2020. The results by sex largely mirror those for the general population. The only noticeable sex difference is that the loss of life expectancy considering year-to-year mortality variations in 2020 was greater for men compared with that for women in most of the 27 countries; this echoes previous findings of a higher mortality of COVID-19 for men^33^. The details are presented in Tables 1 and 2 as well as figures in the Supplementary Information.

## Discussion

Globally, the outbreak of the COVID-19 pandemic has caused a profound mortality increase. A comprehensive understanding of COVID-19’s effects on mortality is needed to better assess the consequences of the pandemic on public and population health. This study provides a new assessment of COVID-19’s effects on mortality in 27 countries, taking into account the year-on-year intrinsic variations of mortality, which have been largely overlooked in previous research. The results indicate that after considering the expected intrinsic changes to life expectancy between 2019 and 2020, the loss of life expectancy caused by COVID-19 amounted to 1.33 year (95% CI = 1.29 years, 1.37 years) at age 15 and to 0.91 years (95% CI = 0.88 years, 0.94 years) at age 65 in the 27 countries. The results suggest that the COVID-19 pandemic has resulted in a considerable increase in mortality in 2020, even in the developed countries where mortality has declined to very low levels, and that the effects of COVID-19 on mortality are more profound than initially expected if the year-on-year intrinsic variations of mortality are considered.

This study better captures the loss of life expectancy caused by COVID-19 and it corrects previously underestimated effects of COVID-19 on human mortality. The results of loss of life expectancy caused by COVID-19 captured in this study are relatively greater than those reported in previous studies in most of the 27 countries studied. For example, the loss of life expectancy *at birth* caused by COVID-19 in the US was previously reported as ranging from 1.18 to 1.87 years without considering the intrinsic year-on-year mortality variations^3,6,10,11^, which were clearly underestimated. The results from this study suggest that the full loss of life expectancy at *age 15* caused by COVID-19 was 2.33 years in the US. The loss of life expectancy at *age 15* caused by COVID-19 in England and Wales (1.48 years), Spain (1.55 years) and Italy (1.48 years) were even slightly higher than the loss of life expectancy *at birth* documented in prior research (1.2 years for men and 0.9 years for women in England and Wales^2^, ranging from 0.9 to 1.18 years in Spain^6,8^ and 1.16 years in Italy^6^). The differences between the loss of life expectancy at *age 15* reported in this study and those *at birth* in prior research are particularly significant in most eastern European countries, such as Poland (1.76 years in our study vs 0.78 years in other studies), Slovakia (1.31 years v 0.40 years) and Bulgaria (1.96 years v 0.71 years)^6^, where life expectancy has increased significantly over the past few decades. These observed differences could be attributed to the use of different data and estimation methods. However, even though not all results of the loss of life expectancy in this study (such as those of Belgium, Denmark and Switzerland) are greater than those reported earlier^6^, this comparison is still reasonable, suggesting that the loss of life expectancy caused by COVID-19 were largely underestimated in previous research, given the neglect of the year-on-year intrinsic changes and variations of mortality. This should be a cause for increased concern among governments, health service providers and the public because the impacts of the COVID-19 pandemic on human mortality might be more significant than previously understood and that the consequences of this devastating pandemic are likely to be long-lasting.

Another remarkable finding of this study is the considerable geographic variations in loss of life expectancy from the COVID-19. Australia, Japan and two northern European countries, Norway and Denmark, experienced less significant loss of (and even a slight increase in) life expectancy from the COVID-19 pandemic in 2020, which was an extraordinary achievement against the backdrop of the COVID-19 pandemic. The success of these four countries in controlling COVID-19 may be attributed to their rapid government response, public trust in government and geographic advantage. First, the government response to COVID-19 in these four countries was swift and decisive. For example, Denmark and Norway had already imposed strict national lockdowns and international travel bans and opened COVID-19 testing to all people before a surge in COVID-19 cases and deaths; this was not seen in many other European nations^34–36^. Additionally, Denmark and Norway also imposed strict interventions for the allocation of scarce hospital resources, which helped decrease stress of the health care system and contributed to the success of lockdown^37^. Similarly, Australia fully closed its international border for more than two years to slow down the spread of the virus, while Japan implemented similar measures, banning international travellers since the first wave of the COVID-19 pandemic emerged until October 2022. Second, public trust in governments may also play a key role. For example, unlike people in other countries with more polarized political sentiments, Danes and Norwegians consistently trust their governments and public health authorities. Thus, social distancing policies were adhered to consistently in Denmark and Norway even when lockdown measures had been announced but were not yet in effect^34,38^. Third, these four countries might have benefited from their geographic locations when controlling the pandemic. For example, the population density of Norway (14.8 persons per square kilometres) and Australia (3.3 persons per square kilometres) are notably lower than many other European countries, such as the UK (280.6 persons per square kilometres) and Italy (205.6 persons per square kilometres)^16^. In addition, Denmark and Norway are also relatively geographically isolated, only sharing borders with one or two countries, compared to many other European countries that are surrounded by other nations in land. Likewise, Australia and Japan are both surrounded by seas, making it relevantly easier control human migration. Low population density and relative isolated geographic locations have likely contributed to a slower spread of COVID-19 in these four countries. The success of Australia, Denmark, Japan and Norway in controlling COVID-19 is encouraging. Their anti-pandemic strategies and experience, particularly swift government response and public trust and cooperation with government’s regulations before the population is adequately vaccinated, have important policy implications for societies elsewhere to effectively reduce the effect of COVID-19 on mortality and also control the current spread of COVID-19.

## Limitations

This study has a few limitations. First, because of data unavailability, this study has not estimated the loss of life expectancy at birth caused by COVID-19; hence, it is unable to analyse COVID-19’s effects on mortality for all age groups. This also limits the comparison between the results of this study and those reported earlier because those earlier studies largely focus on changes of life expectancy at birth. However, given that the case fatality rate of COVID-19 is almost negligible under age 15^21,22^, the loss of life expectancy at age 15 caused by COVID-19 can capture the major effects of COVID-19 on mortality. Second, this study has limited its analysis to within the context of Europe and North America, as well as Australia, Japan and Chile, due to data unavailability for other countries. Therefore, it is unable to capture the effects of COVID-19 elsewhere, particularly in less developed countries where the impacts on human mortality would be more devastating due to generally poorer quality of public health systems than those in developed countries. Life expectancy has increased at a faster pace in the developing world than in the developed world over the past few decades prior to 2020. It is possible that the impacts of COVID-19 on mortality changes in the developing countries might be more significant than in the developed countries covered in this study. Future studies on COVID-19 that consider the intrinsic variations of mortality in the developing world are urgently needed should data become available. Third, assuming there were no other factors breaking down the increasing trend of life expectancy in 2020, changes in life expectancy in 2020 can largely be attributed to the effect of the COVID-19 pandemic. This assumption holds for most of the 27 countries examined in this study since the populations in these countries had seen their age-specific death rate decreasing to varying degrees in the pre-pandemic period. However, for a few countries, such as the US, the life expectancy has already experienced some slight decreases before the pandemic due to an increase in mortality in young age groups^39,40^. Therefore, the loss of life expectancy in 2020 in these countries could be a result of both the previous risk factors driving increased mortality and the effect of the COVID-19 pandemic. Therefore, extra caution is needed when interpreting the loss of life expectancy in these countries. Fourth, in a few countries (e.g., the US), this study projects a relatively considerable decrease in the projected life expectancy at age 65 in 2020. The projected decrease in 2020 is a part of the long-term fluctuating trend, as shown in Figs. 2 and 3. However, this might also be caused by the uncertainties of the projection results of the Lee-Carter model due to overfitting. Fifth, attention needs to be paid to the quality of STMF dataset, particularly regarding its consistency in counting deaths in a few countries. For example, the deaths of residents living abroad are counted in countries such as Bulgaria and Switzerland but not in other countries such as Belgium, Finland and Germany. In addition, countries, such as Belgium, the Czech Republic, Denmark, Norway, Slovakia and Sweden, include all registered deaths but exclude deaths of non-residents (e.g., visitors, non-permanent residents or individuals without registered residency), while some other countries, such as Chile, England and Wales, Greece, Hungary, Italy, Portugal, Scotland and the US, count all death registered regardless of resident status. Moreover, the mortality data in 2020 of six countries (i.e., Chile, France, Greece, Norway, Scotland and the US) from STMF were still considered preliminary (e.g., due to delays in the registration/processing of death events in the medical and civil registration systems) when the data were retrieved for the analysis in this study. Despite these minor inconsistencies in data collection, the STMF dataset has been widely used in COVID-19-related mortality studies^3,14,41^. Sixth, this study does not have the robustness test using other projection methods of mortality; however, given that the Lee-Carter model is well-established in capturing mortality trends, as is the ARIMA random walk model in capturing short-term non-linearities, the projection in this study would not result in the bias normally associated with long-term projection.

## Conclusions

This study offers a new and more comprehensive assessment of the effects of the currently ongoing COVID-19 pandemic on mortality measured by the loss of life expectancy. The results from this study suggest that the full loss of life expectancy caused by the COVID-19 pandemic is greater than previously understood in many countries if the intrinsic year-on-year changes and variations of mortality patterns are considered. Given that the human suffering caused by the pandemic might be more severe than previously understood, much more effort is required to improve policy responses and the cooperation between governments, health service providers and the public to curb the spread of COVID-19 and to increase capacity of public health systems to meet unexpected sudden surges in demand for health services. Strengthened surveillance of infectious diseases and more heightened vigilance around the emergence of pandemics are also suggested. This would help stop the currently ongoing COVID-19 pandemic and also, benefit the prevention and mitigation of future pandemics.

## Supplementary Information

Supplementary Information.

## Data Availability

All datasets used in this study are publicly available. Data from the Human Mortality Database can be found at https://www.mortality.org/, the Eurostat at https://ec.europa.eu/eurostat/web/main/home and the Office for National Statistics (United Kingdom) at https://www.ons.gov.uk/peoplepopulationandcommunity/populationandmigration/populationprojections/datasets/tablea23principalprojectionenglandandwalespopulationinagegroups.
