# Supplementary material for: The effect of the COVID-19 pandemic on life expectancy in 27 countries
Source: Sci Rep. 2023 Jun 1;13:8911. doi: 10.1038/s41598-023-35592-9 (PMC10233553; doi:10.1038/s41598-023-35592-9)
Supplement: Supplementary file 1 — Supplementary Information. [file 41598_2023_35592_MOESM1_ESM.docx]

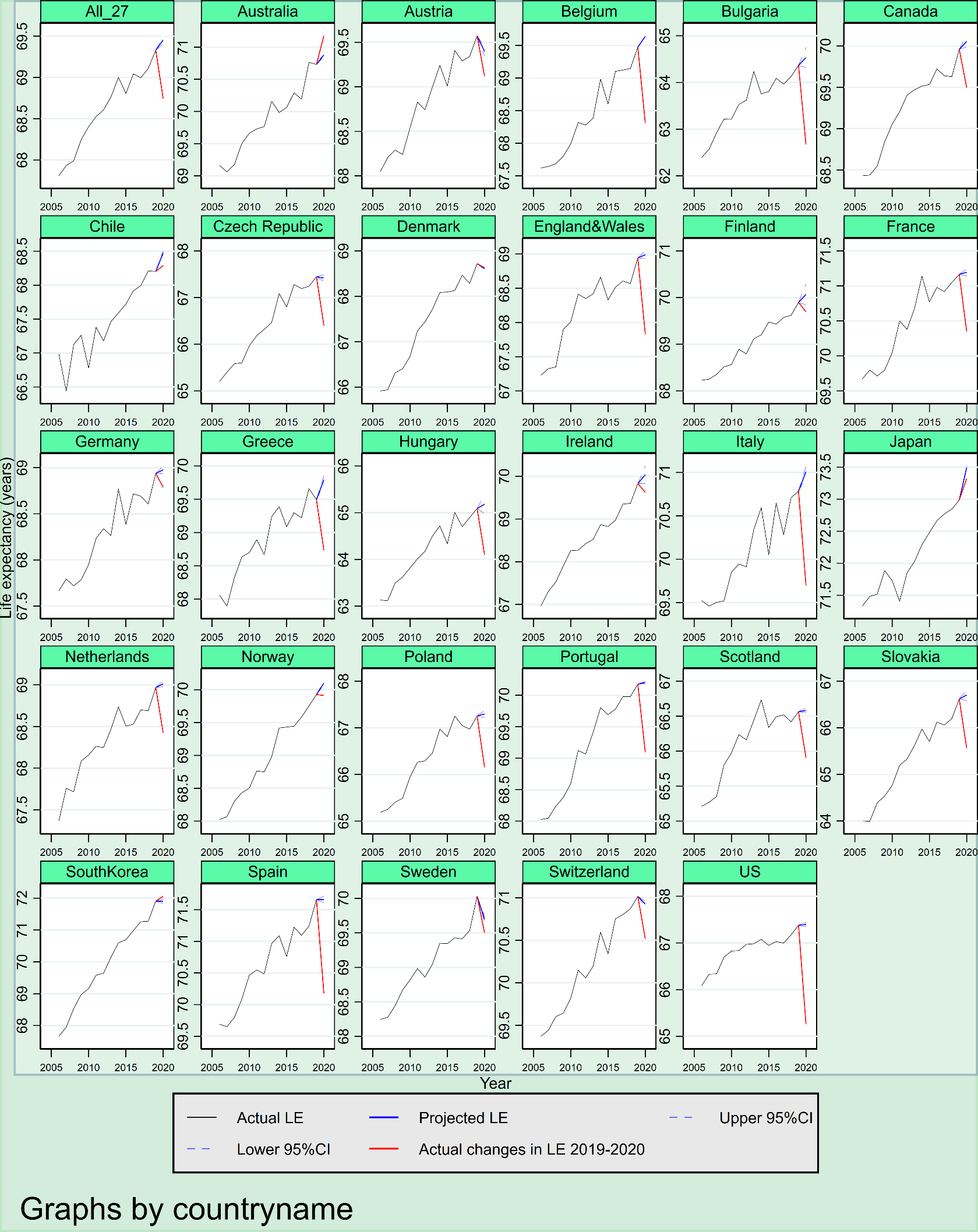


**S-1** Actual and expected life expectancy (LE) at age 15, Female—effects of the COVID-19 pandemic in 27 countries. Actual life expectancy at age 15 before the COVID-19 pandemic is indicated by solid black line and actual life expectancy at 15 from 2019 to 2020 is indicated by red lines. Projected life expectancy at age 15 (i.e., in absence of COVID-19) is indicated by blue line, with 95% confidence intervals (CI) indicated by dashed blue line.


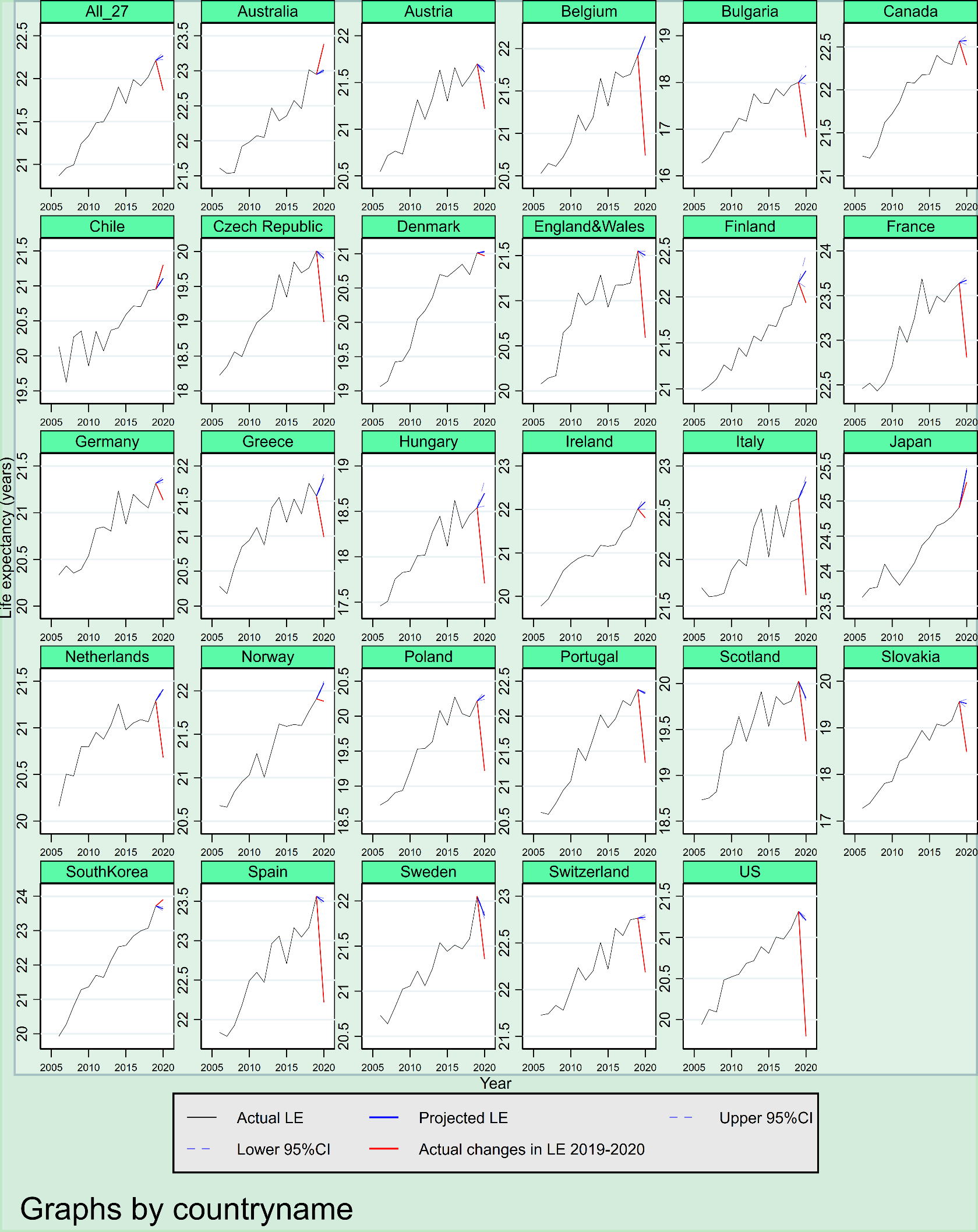


**S-2** Actual and expected life expectancy (LE) at age 65, Female—effects of the COVID-19 pandemic in 27 countries. Actual life expectancy at age 65 before the COVID-19 pandemic is indicated by solid black line and actual life expectancy at 65 from 2019 to 2020 is indicated by red lines. Projected life expectancy at age 65 (i.e., in absence of COVID-19) is indicated by blue line, with 95% confidence intervals (CI) indicated by dashed blue line.


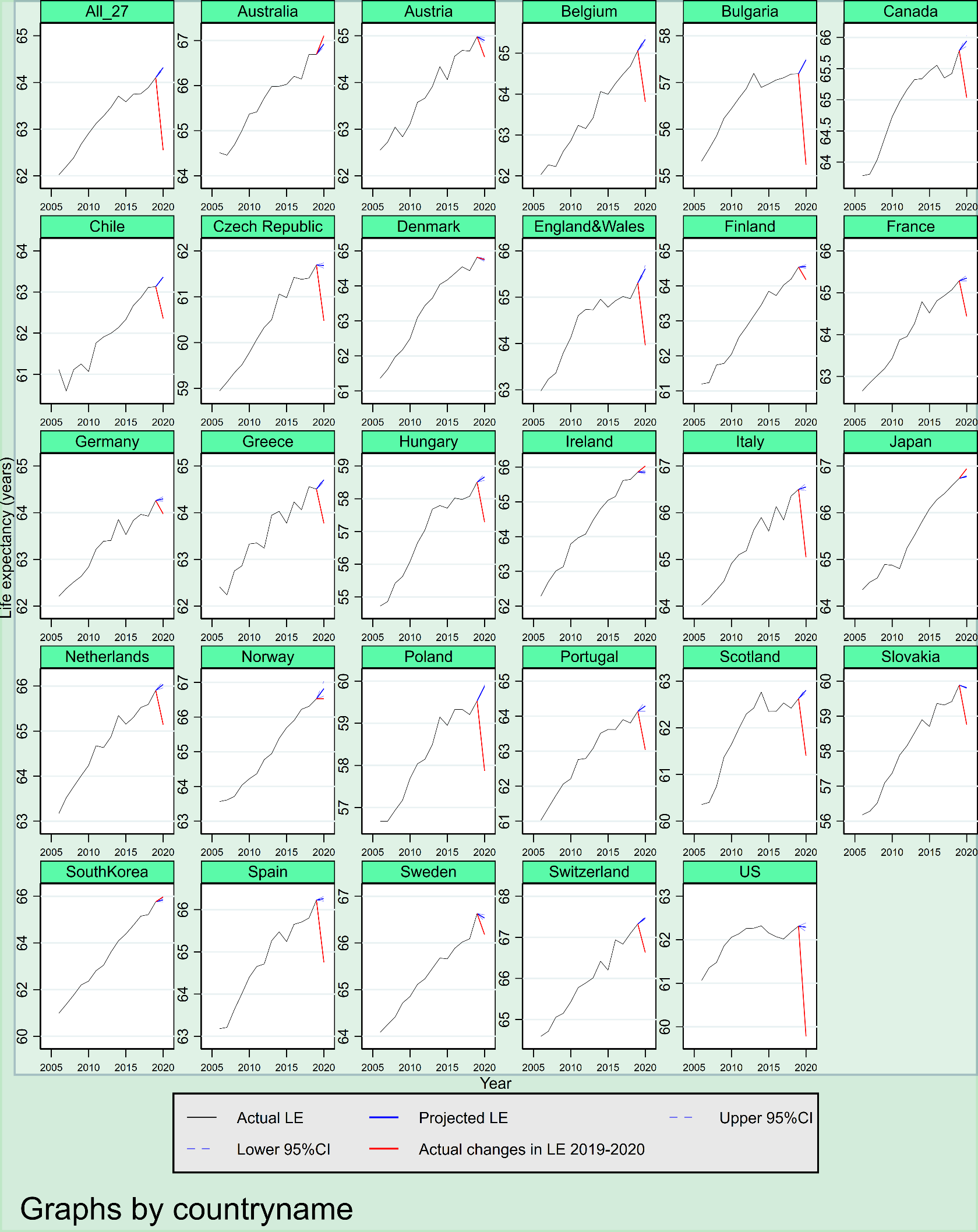


**S-3** Actual and expected life expectancy (LE) at age 15, Male—effects of the COVID-19 pandemic in 27 countries. Actual life expectancy at age 15 before the COVID-19 pandemic is indicated by solid black line and actual life expectancy at 15 from 2019 to 2020 is indicated by red lines. Projected life expectancy at age 15 (i.e., in absence of COVID-19) is indicated by blue line, with 95% confidence intervals (CI) indicated by dashed blue line.


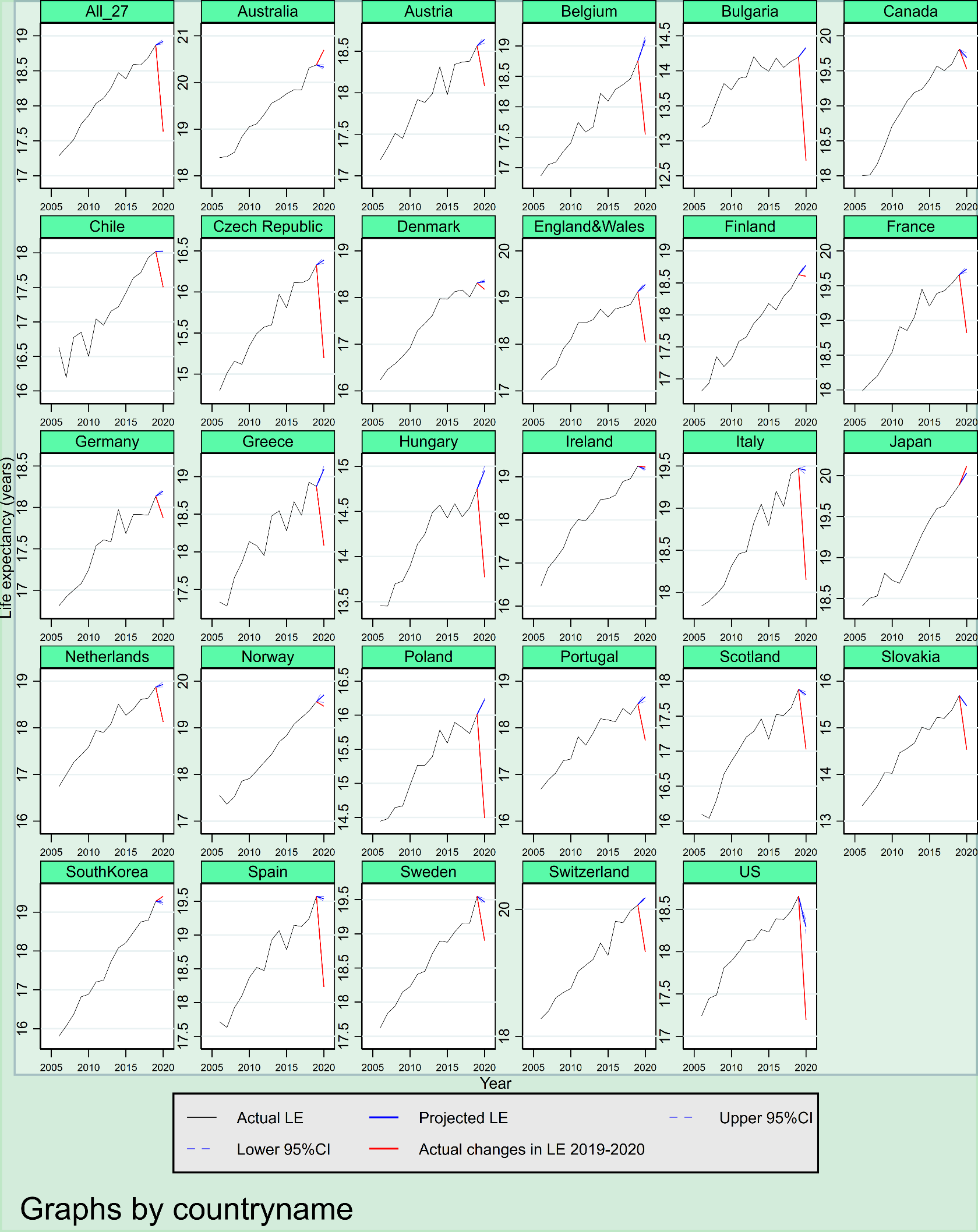


**S-4** Actual and expected life expectancy (LE) at age 65, Male—effects of the COVID-19 pandemic in 27 countries. Actual life expectancy at age 65 before the COVID-19 pandemic is indicated by solid black line and actual life expectancy at 65 from 2019 to 2020 is indicated by red lines. Projected life expectancy at age 65 (i.e., in absence of COVID-19) is indicated by blue line, with 95% confidence intervals (CI) indicated by dashed blue line.

**Rules of for determining the values of (p, d, q) in ARIMA models**

- The value of d is determined by examining how the time series has become stationary, with the value selected based on at which order differencing the immediate lag has gone on the negative side in the autocorrelation plot.
- The value of p is determined based on which lag can cross a significance limit in the partial autocorrelation function plot.
- The value of q is determined by seeing how many the lags are out of the significance limit in the autocorrelation plot.
- After choosing the values of (p, d, q), we also tried the options using lower values of p and q. For example, we also tested (1,0,1), (2,0,1) and (1,0,2) if the values of (p, d, q) were determined as (2,0,2).
- The optimal group is selected based on three criteria: (1) whether the model is significant, (2) whether the sum of Akaike's Information Criterion (AIC) and the Schwartz Bayesian Information Criterion (BIC) is lowest and (3) whether q+p ≤5.

**Construction of Life Table**

Life tables were constructed following the method proposed by Chiang ^20^, outlined as follows.

Let ${}_{n}m_{x}$ represent the crude death rate for the age group (*x*, *x*+*n*), where *x* denotes age and *n* denotes age interval, and ${}_{n}a_{x}$=$\frac{n}{2}$, where ${}_{n}a_{x}$is the average fraction of the age group (*x*, *x*+*n*) lived by individuals dying at any age included in the age group.

First, probability of death ${}_{n}q_{x}$can be computed as below:

${}_{n}q_{x}=\frac{n*{}_{n}m_{x}}{1+(1-{}_{n}a_{x})*n*{}_{n}m_{x}}$ (Equation 1).

For the highest age group,

${}_{\infty}q_{80}=1$(Equation 2).

Based on${}_{n}q_{x}$, indicators of${}_{n}p_{x}$, ${}_{n}d_{x}$and${}_{n}l_{x}$, representing the probability of survival, the number of deaths, and the number of surviving persons for the age group (*x*, *x*+*n*), respectively, can be obtained through the following equations:

${}_{n}p_{x}=1-{}_{n}q_{x}$(Equation 3),

${}_{n}d_{x}=l_{x}*{}_{n}q_{x}$(Equation 4),

$l_{x+n}=l_{x}-{}_{n}d_{x}$(Equation 5).

For age 0,

$l_{0}=100,000$ (Equation 6).

Then, person-years lived between age *x* and *x*+*n*, ${}_{n}L_{x}$, can be computed by Equation 7:

${}_{n}L_{x}=n*l_{x+n}+{}_{n}a_{x}*{}_{n}d_{x}$(Equation 7).

For the highest age group,

${}_{\infty}L_{80}=\frac{l_{80}}{{}_{\infty}m_{80}}$(Equation 8).

Person-years lived above age *x*, $T_{x}$, is computed as follows:

${}_{n}T_{x}=\sum_{i=x}^{\infty} {}_{n}L_{x}$(Equation 9).

Finally, life expectancy at age *x*, $e_{x}$, can be estimated by the following equation:

$e_{x}=\frac{T_{x}}{l_{x}}$(Equation 10).
